# Sphero-Conical Modeling for the Estimation of Very Long Baseline Interferometry Invariant Point

**DOI:** 10.3390/s22207937

**Published:** 2022-10-18

**Authors:** Tae-Suk Bae, Chang-Ki Hong

**Affiliations:** 1 Department Geoinformation Engineering, Sejong University, Seoul 05006, Korea; 2 Department Geoinformatics Engineering, Kyungil University, Gyeongsan 38428, Korea

**Keywords:** very long baseline interferometry, invariant point, international terrestrial reference frame, local tie, total least-squares method

## Abstract

A geodetic reference frame is a fundamental element in geoinformation fields such as autonomous navigation and digital twins. The international terrestrial reference frame is established and maintained using several space-geodetic techniques, including very long baseline interferometry (VLBI) and satellite laser ranging (SLR). For several decades, geodesists have been devoted to connecting these two sensors at a site (local tie). The reference point of the VLBI antenna and SLR telescope, called invariant point (IVP), should be precisely determined to connect these two solutions. We developed an innovative integrated model to estimate the IVP, which is composed of spherical and conical models, depending on the rotational axis. In this model, all target points in 3D spaces were directly connected to the IVP; thus, the stability and robustness of the system were secured. Furthermore, all inherent errors in the coordinates were predicted by applying the total least-squares approach.

## 1. Introduction

The international earth rotation and reference systems service (IERS) has contributed to establishing and maintaining the international terrestrial reference frame (ITRF) by analyzing and combining data from five different space-geodetic systems such as global navigation satellite system (GNSS), satellite laser ranging (SLR), lunar laser ranging, very long baseline interferometry (VLBI), and Doppler orbitography and radiopositioning integrated by satellites [1,2,3,4].

The “co-located sites” are the stations where more than one of the five instruments are in operation simultaneously. They play a pivotal role in the combination of data observed by different physical sensors located away from each other via a local tie vector. A local tie vector represents a three-dimensional (3D) baseline vector between the reference points of two space-geodetic instruments [5,6]. Therefore, a precise geodetic reference frame, that is, the ITRF, should be determined with a high level of accuracy. Multiple VLBI radio telescopes are used for the determination of precise baseline vectors between the two points on the Earth or in space. The baseline vector’s starting and ending points refer to the radio telescope’s reference point (or invariant point, IVP), which usually deviates from the phase center of the radio telescope. The IVP of the VLBI antenna is defined as the intersection point between the primary (azimuth) and secondary (elevation) axes of the antenna. If these axes do not intersect, the reference point is defined as the projected point on the primary axis [7].

In general, an IVP is physically located inside the antenna structure, and its coordinates cannot be estimated directly from ground surveys. Therefore, the indirect approach has been widely used for the determination of the IVP by observing targets attached outside the VLBI antenna during rotational sequences [8,9].

The coordinates of the targets, together with their variance–covariance information, were obtained from ground surveys by applying the least-squares method. Then, the IVP estimation procedure is performed using the geometric relationship between the targets and IVP. The 3D circle fitting method is commonly used to estimate the IVP. The trace of a target on the antenna rotating about an independent axis can be expressed as a 3D circle that can be defined using seven parameters, including three parameters for the center of the circle, three parameters for the unit normal vector, and one parameter for the radius of the circle [8,10,11,12,13,14]. Slightly different approaches have also been introduced by imposing various types of geometric conditions [5,15].

The trace of each target due to the horizontal rotation presents a full circle in normal surveying conditions, which increases the stability for parameter estimation. However, the trace of a target by the vertical motion in elevation makes only a quarter circle because of the limited motion of the rotational axis. Consequently, it leads to an unstable linear system being solved. Moreover, each rotational sequence of the elevation axis yields seven parameters for modeling each arc. The increase of rotational sequences requires more parameters to be estimated, which is one of the disadvantages of the 3D circle fitting method.

In this study, we propose a new model for the determination of the IVP from the coordinates of targets estimated based on terrestrial observations. The basic concept of the new approach is that the trace of horizontal (azimuthal) rotation becomes a 3D circle in space. However, the motion in elevation creates a cone shape with an apex at the IVP, where the slant distance of the cone is the only unknown for a vertical target. Therefore, a new approach, called the sphero-conical model, connects all target positions in the 3D space to a common IVP, which significantly reduces the unknowns compared to the conventional methods. In other words, the primary unknowns are the coordinates of the IVP and an additional parameter for each target, representing either horizontal or vertical motions.

Setting aside the small number of observations, the conventional model based on a 3D circle may lead to an ill-conditioned linear system when circle arcs are incomplete; as a consequence, the quality of the estimated parameters will be degraded [5]. However, the new sphero-conical model can overcome the ill-conditioned problem and obtain efficiency in terms of the redundancy of the linear system to be solved, which is the most significant contribution of this study.

It should be noted that the coordinates of the observed targets are observations; hence, they have inherent random errors. Therefore, the adjustment computation must be performed by estimating not only the IVP, but also the coordinates of the targets on the antenna. The total least-squares (TLS) approach was adopted in this study because it minimizes random errors in all data variables [16].

In the following sections, detailed descriptions of the new sphero-conical model for IVP estimation and the numerical test results with in situ measurements are provided.

## 2. General Description

### 2.1. Project Overview

Although the space-geodetic sensors are located at the same station, they are physically different from one another. Therefore, the connection between two different sensors can only be accomplished by ground surveying, such as slant distance and horizontal and/or vertical angles, using total stations and/or theodolites. For IERS to accurately combine solutions obtained from both GNSS and VLBI techniques, the coordinate vector between their respective antennas (called the local tie vector) must be precisely determined. As the VLBI antenna rotates in any direction, its point of rotation remains fixed in location, which is the antenna’s IVP. Thus, we must determine accurate coordinates of the VLBI IVP.

**Figure 1:**
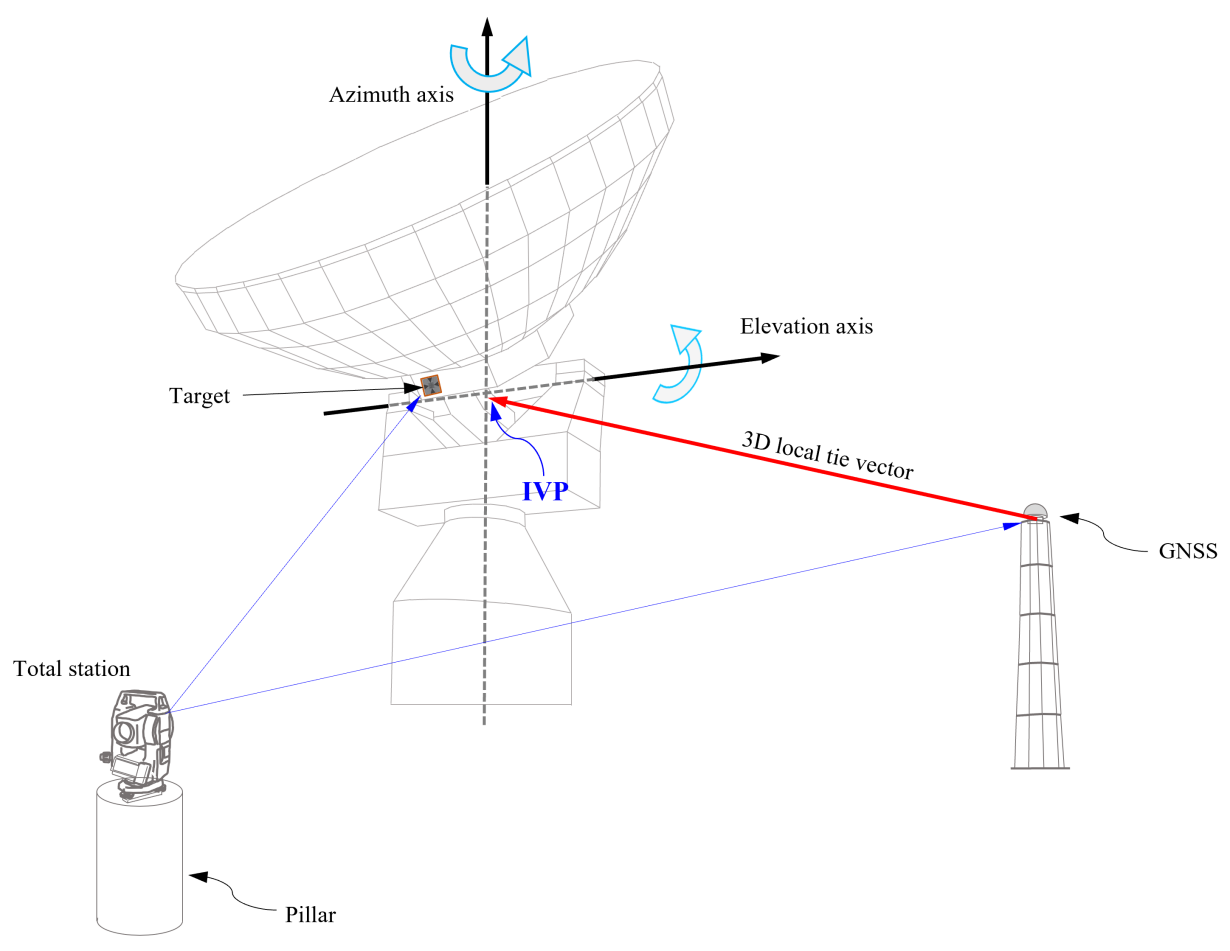
The schematic drawing of VLBI IVP estimation process. The IVP is the geometric center of the VLBI antenna, and the local tie vector with respect to the GNSS is the primary concern. The IVP is estimated based on the ground surveying measurements (slant distances, horizontal/vertical angles, etc.) from total station mounted on the permanent pillars.

The IVP location (coordinates) with respect to the continuously operating reference station (CORS) antenna was determined from measurements made during local ground surveying (see Figure 1). However, because the IVP is physically located inside the antenna structure, its coordinates cannot be directly estimated from ground surveys. Thus, a mathematical model should be developed to connect the IVP and the targets attached outside the antenna.

The main task of the project is to connect the GNSS and VLBI antennas by measuring and adjusting the ground survey data. In the following, we often refer to these two antennas, or their locations, as “GNSS antenna” (or just GNSS) and “VLBI antenna” (or just VLBI), respectively. We developed a new model that relates all target coordinates to a single IVP; thus, every single point in the 3D space is connected to the IVP. In addition, the set of coordinates for each target is obtained from the ground survey; thus, each component of the coordinate set has an intrinsic error that should be considered in the adjustment process. Therefore, we applied the TLS approach based on the Gauss–Helmert model (GHM) to account for all error components in the target coordinates [16,17,18].

### 2.2. Estimation Procedure

**Figure 2:**
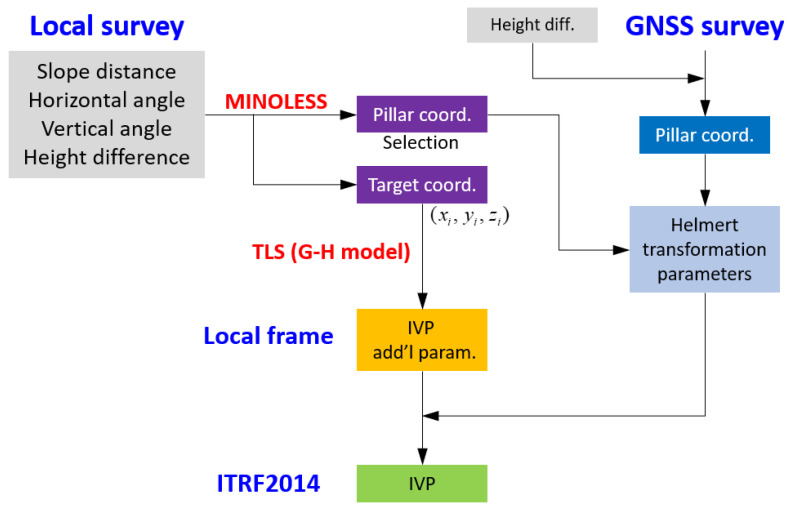
Flow chart to estimate the VLBI IVP [24]. The ground surveying and the GNSS campaign are conducted under two reference frames. The IVP estimated in local frame is transformed into the global frame via the conventional Helmert transformation.

Figure 2 shows the overall plan for estimating the VLBI IVP based on a local ground survey. All survey measurements were obtained from the permanently equipped pillars surrounding the VLBI antenna. Four different types of observations were considered in this study: slope distance and horizontal/vertical angles using a total station, along with the height difference from traditional spirit leveling. Once ground surveying is completed, we can apply the (partial) minimum norm least-squares solution (PMINOLESS/MINOLESS) to minimize the distortion of the (local) reference frame.

The VLBI IVP, coordinates of the pillars, and targets were estimated based on the mathematical model developed in this study. The estimation process is based on the TLS within the GHM, in which the coordinates of the pillars/targets are estimated simultaneously.

In the final step, the VLBI IVP should be represented in the global reference frame (e.g., ITRF2014), which can be performed using the Helmert transformation typically performed in geodetic reference frames [19,20,21,22,23]. The transformation parameters were estimated using pillars. The GNSS campaign should be conducted to obtain coordinates aligned to the ITRF. Finally, the local tie vectors should be represented with respect to each other (in this study, we used the GNSS antenna as a reference point), along with the full variance–covariance matrix between the VLBI IVP and GNSS reference point.

### 2.3. Ground Surveying

Ground surveying is generally conducted in a local frame, whose vertical axis is naturally determined from the local plumb line. Although the origin of the local frame can be set up anywhere within the site, CORS SEJN was chosen as the origin of the local reference frame. This is because it should eventually be aligned to the ITRF to be combined with other space-geodetic solutions, which is performed using the GNSS campaign at local pillars, including CORS SEJN. Therefore, the local tie vector is represented with respect to the GNSS pillar (SEJN). The orientation of the local reference frame will be defined using the local geodetic coordinate system of the CORS (SEJN); thus, there might be an offset called the “deflection of the vertical” between the geodetic vertical direction and the local gravity vector [25]. However, the magnitude is very small at the project site (approximately 6 arcseconds in the absolute sense), and it should be absorbed into the Helmert transformation parameters while transforming the local tie vector into the global reference frame.

All possible slant distances and horizontal/vertical angles between the pillars were observed with a total station (an electronic theodolite integrated with an electronic distance-measuring instrument) [26]. Because it is not feasible to mount a glass-prism reflector at the antenna reference point (ARP), only the horizontal and vertical angles were measured from the pillars to the ARP of the CORS. Moreover, it is not possible to locate a center to the target on the ARP; thus, the horizontal and vertical angles are measured to the bottom-left and bottom-right of the antenna, and their average is used as a measurement of the ARP.

To determine the local tie vector in the ITRF, the coordinates of the eight pillars and the CORS (SEJN) ARP should be estimated in both the local and global frames. A GNSS campaign was conducted by simultaneously collecting data from GNSS receivers mounted on all eight pillars and the CORS. Data were collected at a 30 s rate over seven 24 h sessions for one week. The GNSS data collected at the pillars were processed together with those from 51 international GNSS service core stations worldwide, resulting in a set of GNSS vectors aligned to ITRF 2014. The resulting vectors, along with their covariance matrices, were used as observations in the subsequent network adjustment.

### 2.4. Survey of Targets

A total of 13 targets were mounted on the VLBI antenna, which consisted of seven prisms and six sheet targets at different locations [14,27]. All possible distances from each of the five pillars around the VLBI antenna to the targets were measured using the total number of stations. The elevation angle was fixed at $90^{\circ }$ for all measured horizontal angles. The total station measures all available targets during the rotational sequences of the azimuth axis (i.e., $30^{\circ }$ increments in the rotational angle).

Likewise, all possible horizontal and vertical angles between the pillars and targets on the VLBI antenna were measured together. The coordinates of the targets and their covariance matrix were estimated via a least-squares adjustment of data. Minimally constrained adjustments were first performed using PMINOLESS to minimize the norm of the parameter vector [28].

## 3. Mathematical Model for IVP Estimation

**Figure 3:**
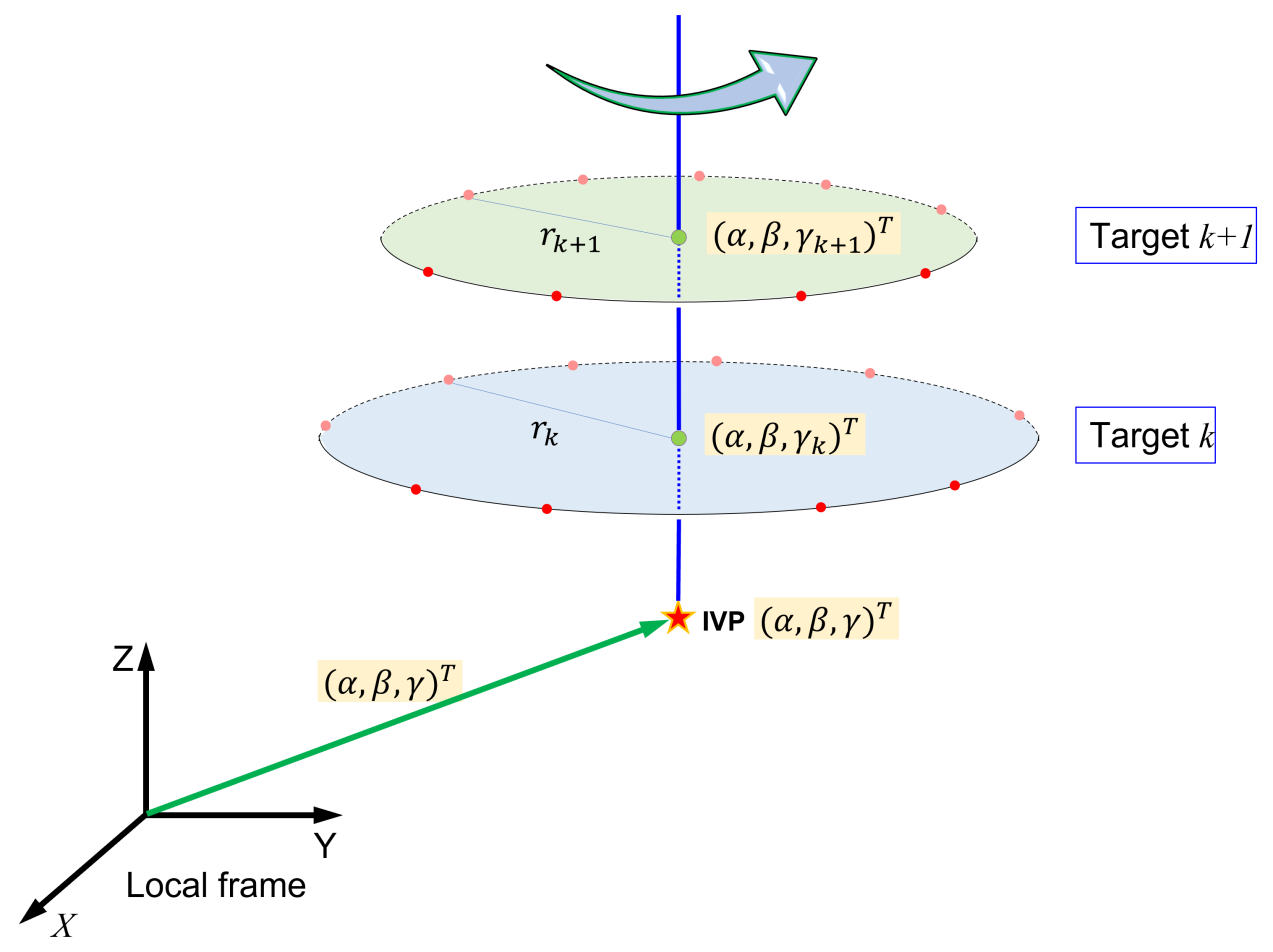
The azimuthal trace of two targets. The horizontal planes generated by each target share the common normal vector as well as the horizontal coordinates of the center.

**Figure 4:**
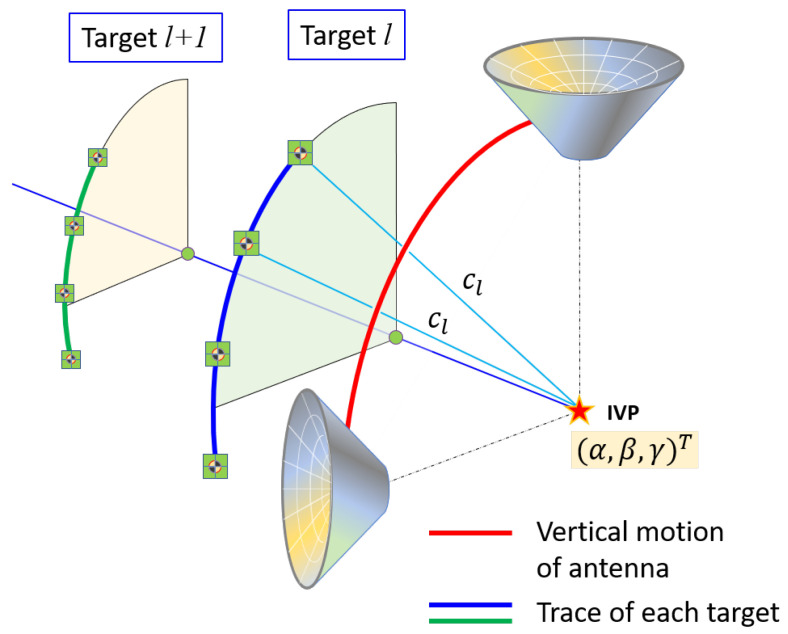
Rotation in elevation (i.e., in a vertical plane). Each quarter circle is part of a cone, with the apex at the IVP.

All targets were attached “outside” the VLBI antenna (the opposite side of the concave area). Two different motions are possible for the VLBI antenna: (a) azimuthal rotation through $360^{\circ }$ (see Figure 3), and (b) rotation in elevation, that is, in a vertical plane by $90^{\circ }$, from the zenith to the horizon at any azimuth (Figure 4). Theoretically, by combining the azimuthal rotation and motion in elevation, we can measure as many locations as possible in 3D for each target, resulting in an almost half-sphere that comprises the antenna movement. However, targets on the antenna surface are usually not visible for zenith angles greater than $40^{\circ }$ because of self-obstruction; thus, the actual number of measurements will be less than expected.

### 3.1. Azimuthal Rotation of the Antenna

Assume that the local and VLBI-specific frames are aligned with each other. The azimuthal rotation of the targets is depicted in Figure 3, where the traces of the two targets are shown to generate concentric circles of different heights, the centers of which have horizontal coordinates that coincide with those of the VLBI IVP. The radii of the circles differ for each target depending on the location of the antenna.

The radius of the horizontal circle ($r_{k}$) and height of the plane ($\gamma_{k}$), both for target *k*, are highly correlated. Therefore, it is desirable to estimate only the radius of the circle, which is related to the horizontal components. The mathematical model for the circle on the horizontal plane traced by target *k* through points $\left(x_{i},y_{i}.z_{i}\right)$ at height $\gamma_{k}$ can be expressed as

(1) $$R_{k}\left(\alpha ,\beta ,r_{k}\right)={\left(x_{i}-\alpha \right)}^{2}+{\left(y_{i}-\beta \right)}^{2}-r_{k}^{2}=0,$$

where $r_{k}\;\left(k=1,\cdots ,n_{k}\right)$ is the radius of the circle centered at (α,β), which is the horizontal component of the IVP, and $n_{k}$ represents the number of targets to be estimated. Thus, as explained earlier, all targets with azimuthal rotation are related to the horizontal component of the IVP, which provides a large degree of freedom to estimate it.

### 3.2. Rotation of Targets in Elevation

The targets were generally located away from the vertical plane of rotation through the IVP. Because rotation through a vertical plane causes the vertical axis of the antenna to move from the zenith to the horizon, the targets will trace a quarter circle with its center on the rotation axis (Figure 4). The radii of the traced circles will be different for each target, depending on the orthogonal distance between the vertical plane it traces and the parallel vertical plane that contains the IVP.

**Figure 5:**
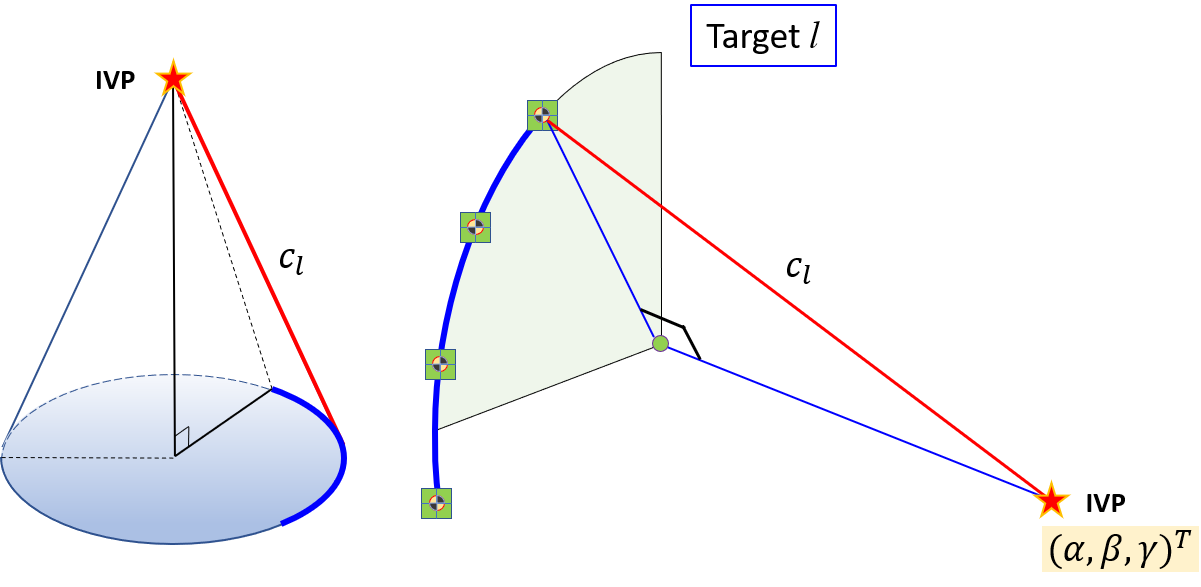
The rotation of targets in elevation represented by a cone with the apex at the IVP. The slant height $c_{l}$ is the individual unknown for each vertical target, while the coordinates of the IVP are common for all targets.

The center of each quarter circle is located on the same horizontal plane that passes through the IVP. However, the radius of the vertical quarter-circle is highly dependent on the height component of the IVP (γ), which makes the system unstable to estimate. Instead of estimating the radius and horizontal components of the quarter-circle, we estimated the slant height of a cone with the apex at the IVP (see Figure 5). Therefore, the mathematical model for the vertical rotation (or rotation in elevation) of target *l* can be set up to represent the distance between the IVP and the observed targets directly, as follows:

(2) $$C_{l}\left(\alpha ,\beta ,\gamma ,c_{l}\right)={\left(x_{i}-\alpha \right)}^{2}+{\left(y_{i}-\beta \right)}^{2}+{\left(z_{i}-\gamma \right)}^{2}-c_{l}^{2}=0,$$

where $c_{l}$ is the slant height of the cone formed by the vertical rotation about the apex at the IVP.

Because the horizontal coordinates of the IVP, i.e., (α,β), are sufficiently controlled by observations made during horizontal rotation, it is expected that the vertical coordinate (γ) of the IVP and the slant height of the cone ($c_{l}$) are separable from each other and determined appropriately.

The complete “sphero-conical” model can be found in Appendix A.

## 4. Estimation of IVP Using TLS

In general, only the dependent variables were considered to have random measurement errors in the conventional least-squares adjustment, whereas the independent variables were treated as error-free. By TLS, we refer to a least-squares estimator that minimizes the random errors in all data variables [16,17,29], sometimes called “general least squares” [17,30].

The coordinates of the targets mounted on the VLBI antenna can be estimated from surveying data such as slope distances, and horizontal/vertical angles; thus, all three components have errors, together with the covariance between parameters. Therefore, we must consider applying the TLS method to estimate the IVP coordinates based on the coordinates of the targets.

If the target is related to one rotation only, either horizontal or vertical rotation, the corresponding row (upper or lower) in $w_{i}$, $A_{i}$, and $B_{i}$ should be considered accordingly. (See the detailed derivation of the IVP estimation within GHM in Appendix B). Collecting all the related equations leads to the following GHM [16,29,31,32]:

(3) $$w=A\;\xi +B\;e,\;e∼(0,\sigma_{0}^{2}P^{-1}),$$

where w is the observation vector, *A* is the design matrix, ξ is the parameter vector, *B* is the transformation matrix, e is the observation error vector, $\sigma_{0}^{2}$ is the variance component, and *P* is the weight matrix for the observation. The combined unknown parameters can be explicitly represented by

(4) $$\begin{array}{cc} \xi & ={\left[\begin{array}{ccc} \xi_{1}^{T} & \xi_{2}^{T} & \xi_{3}^{T} \end{array}\right]}^{T}\\ \xi_{1} & ={\left[\begin{array}{ccc} \alpha & \beta & \gamma \end{array}\right]}^{T}\\ \xi_{2} & ={\left[\begin{array}{ccc} r_{1} & \cdots & r_{n_{k}} \end{array}\right]}^{T}\\ \xi_{3} & ={\left[\begin{array}{ccc} c_{1} & \cdots & c_{n_{l}} \end{array}\right]}^{T}. \end{array}$$

As can be seen in Equation (4), the unknowns are grouped by their types ($\xi_{1},\xi_{2},\xi_{3}$); the first group (α,β,γ) represents the coordinates of the IVP, as explained above, and the second and third groups are related to the horizontal and vertical motions, respectively, while $n_{k}$ and $n_{l}$ are the corresponding numbers of targets. $\xi_{2}$ and $\xi_{3}$ are the nuisance parameters, which are the radii of the horizontal circles and the slant heights of the cones, respectively, and are of no primary concern. However, these parameters must be estimated simultaneously to complete the model with solid and robust solutions.

## 5. Experiments and Discussion

To evaluate the completeness and/or compatibility of the mathematical models, numerical verification was performed using the survey results from March 2019 [33]. The initial coordinates are given in Table 1, along with the IVP estimated using conventional methods (the last row of the table). The local coordinate system was established by using VP01 as the origin, and VP02 was oriented to the ***x***-axis (east direction), where height information was adopted from a spirit leveling survey (orthometric height). The estimated IVP values were based on the 3D circle fitting of the conventional LESS for each target, which was averaged to generate the final solution.

The observations are the coordinates (x,y,z) of each target in 3D. The total number of observations was 423 points, of which 241 points were related to azimuthal rotations and 182 points were used for the vertical motion model.

**Figure 6:**
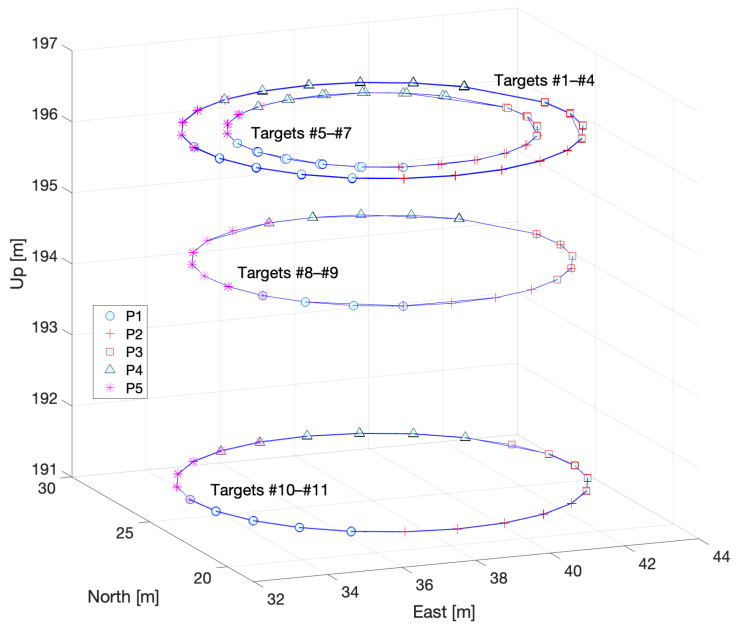
The trace of azimuthal rotation of targets. Each symbol represents the related pillars (P1 to P5) on which the instrument was installed for ground surveying.

**Figure 7:**
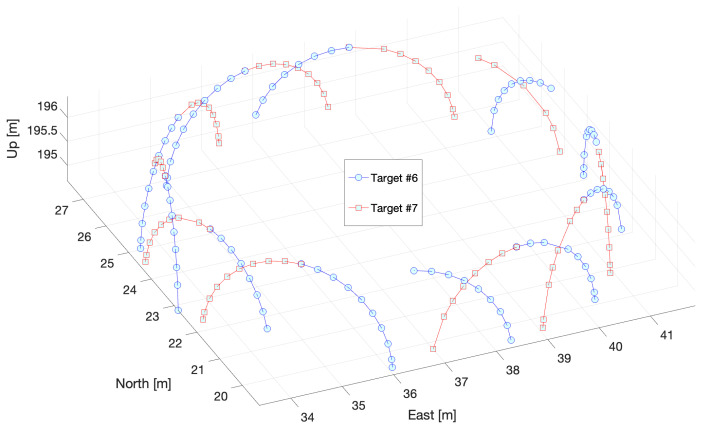
The trace of vertical motion of targets (only two targets are involved). Two targets are located on the opposite sides of the structure under the antenna.

Figure 6 shows a trace of the azimuthal rotation of each target, where the symbols represent the pillars related to the determination of the coordinates. As shown in the figure, only partial directions are covered by a specific pillar; thus, full coverage ($360^{\circ }$ around the antenna) is completed by five pillars. For vertical motion, only two targets were used, that is, #6 and #7, as shown in Figure 7. The vertical motion of the antenna was conducted every $30^{\circ }$ in the azimuth.

Table 2 describes the parameters used in the model, where the IVP is expressed using only three parameters. Eleven parameters are related to the radii of the horizontal circles ($r_{k}$) by the azimuthal rotation of each target, resulting in the same number of unknowns. Similarly, distance ($c_{l}$) explains the slant heights of a cone traced by the vertical motion of two targets with the apex at the IVP. It is remarkable that only five parameters, i.e., the coordinates of the IVP and the slant height of two cones, are used for the trace of vertical targets, regardless of the number of azimuthal sequences. Contrary to the proposed model, a total of 168 parameters (2 targets × 7 parameters for 3D circle × 12 azimuthal rotation sequences, assuming $30^{\circ }$ interval in azimuth) are required to implement the conventional 3D circle fitting algorithm.

Numerical results show that the TLS method with the GHM works well in this experiment. As can be seen in Table 3, the criterion of $10^{-5}$ m on the 2-norm of the entire parameter update was satisfied after four iterations. More specifically, the model converges after the first iteration and corrections to the parameters became small immediately, and the quality of fit ($\Omega ={\tilde{e}}^{T}P\tilde{e}$) as well as the variance component estimates (${\hat{\sigma }}_{0}^{2}$) change very little after two iterations.

The estimated parameters, along with the precision of the estimates are summarized in Table 4. The initial approximation for each parameter was used to linearize the nonlinear functional relationship of the GHM.

As can be seen in the table, the radii of each motion for the targets were estimated with a precision of <1 mm. For the IVP, the horizontal components were estimated to be even better than the vertical components, in terms of the dispersion of the estimates. This is because all horizontal and vertical targets were related to the horizontal component (α,β), whereas only two targets were involved in the vertical component (γ).

Although the 3D coordinates of the targets were used in this experiment, raw ground measurements, such as slant distance, horizontal/vertical angles, and height differences, can also be used to estimate the IVP. Furthermore, as mentioned above, the coordinates have intrinsic errors; thus, the TLS approach should be applied to simultaneously predict the errors in the coordinates of each target.

**Figure 8:**
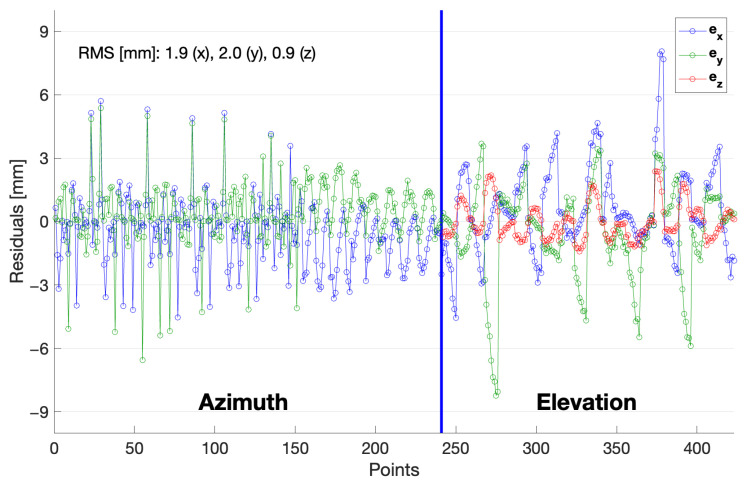
Residuals of each observation after TLS adjustment. The *z*-component is not involved in the azimuthal rotation, and the periodic large residuals are related to the vertical motion at specific azimuth.

Figure 8 shows the residuals of all the components with a root-mean-squared error of 2 mm or less. Because the z-component was not used in the horizontal rotation model, no residuals were calculated for $e_{z}$ in azimuthal rotation. As can be seen in Figure 8, several successive measurements have larger residual errors, particularly for the measurements from motion in elevation (up to about 150 points in azimuthal rotation as well). This seems to be attributed to either rotation at a specific azimuth or measurements at a specific pillar, which should be investigated further.

**Figure 9:**
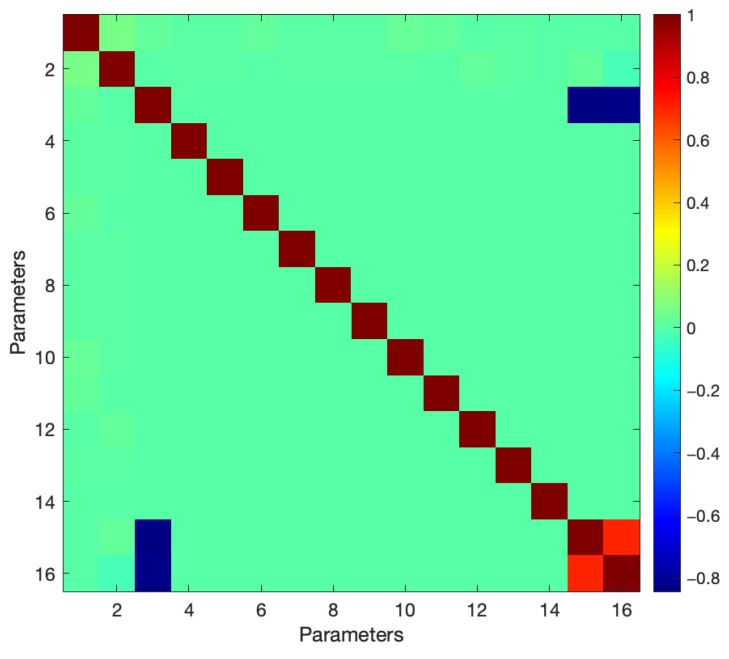
Correlation between parameters after TLS adjustment. All correlations are resolved except the one between the UP component (γ) and the slant heights of the vertical targets.

To verify the stability and separability between the parameters of the system, all correlations between the parameters listed in Table 4 were investigated by calculating cross-correlations (see Figure 9). As seen in Figure 9, all correlations were fully resolved except for the one between the UP component (γ) of the IVP and the distance parameters ($c_{l}$) to the target related to the vertical motion, which is expected from the geometry of the antenna.

## 6. Conclusions

In this study, we proposed a sphero-conical model and successfully applied it to estimate the VLBI IVP, which is the primary concern in establishing and maintaining global geodetic reference frames based on several space-geodetic techniques. It is very important to note that only three unknowns are necessary to estimate the IVP (and one additional parameter per target). In other words, all measured target positions are directly connected to the IVP; thus, no single target is in vain during several steps of 3D circle fitting, as in conventional methods. The total number of parameters is significantly reduced in the proposed method, especially for the traces of vertical targets (e.g., only 3% of parameters are necessary compared to the conventional 3D circle fitting algorithm). Thus, this model can increase the separability between parameters and the numerical stability of the system.

More importantly, the newly developed sphero-conical model can overcome the limitation of geometry arising from the VLBI antenna rotation. Especially, the conventional model should estimate the 3D circle based on partial data only (e.g., a quarter-circle or less), resulting in considerable instability in the estimation process. As explained earlier, ground survey measurements, such as slant distances and horizontal/vertical angles, can be directly used in a sphero-conical model. In addition, when combined with TLS within the GHM, we can estimate the errors in all components of the target coordinates in the 3D space.

It should be noted that the vertical offset of the antenna axis must be considered, although it was assumed to be parallel to the plumbline in this study. There might be two types of offsets: (a) the horizontal offset of the vertical axis or (b) the inclination of the vertical axis. These offsets should be modeled to complete the estimation of the invariant point [17]. Because several types of ground survey measurements with possibly different levels of precision are involved in the adjustment, the variance component model needs to be considered instead of a single variance component [27,34,35,36].

Lastly, two motions (azimuthal rotation and motion in elevation) were modeled differently in this study: either the radii of the horizontal circles in the 3D space or the slant distances of the cones with the apex at IVP. However, a unified, homogeneous model that incorporates two motions makes the system simpler and more consistent, which will be discussed in future research.

## Figures and Tables

**Table 1:** Initial coordinates of each pillar/CORS along with the previous results of IVP estimation.

Station	*x* [m]	*y* [m]	*z* [m]
VP01	0.0000	0.0000	177.9075
VP02	46.5314	0.0000	180.8358
VP03	64.9459	15.4701	180.9527
VP04	38.0156	65.5444	180.5815
VP05	1.0564	50.7578	185.5912
SEJN	7.1632	−26.3145	181.1960
*est.* IVP	37.6278	23.4907	194.5977

**Table 2:** List of parameters used in this experiment.

Parameter	Notation	Description
1–3	(α,β,γ)	IVP where $\left(\alpha^{0},\beta^{0},\gamma^{0}\right)=\left(37.6,23.5,194.0\right)$ [m]
4–14	$r_{k}$	Radii of horizontal circles
15–16	$c_{l}$	Slant heights of a cone traced by vertical motion, with the apex at IVP

**Table 3:** Outputs of each iteration process (primary parameters only).

Iteration#	α	β	γ	$\Omega ={\tilde{e}}^{T}P\tilde{e}$	${\hat{\sigma }}_{0}^{2}$	$|\hat{\xi }|$	$|{\hat{\xi }}_{\alpha ,\beta ,\gamma }|$
Initial	37.6000	23.5000	194.0000	–	–	–	–
1	37.6300	23.4885	194.5904	789.1273	1.9389	0.7181	0.5913
2	37.6301	23.4885	194.5904	829.3425	2.0377	0.0466	0.0001
3	37.6301	23.4885	194.5904	829.3493	2.0377	0.0002	0.0000
4	37.6301	23.4885	194.5904	829.3493	2.0377	0.0000	0.0000

**Table 4:** Estimated parameters, along with the initial approximations.

ξ	$\xi_{i}^{0}$ [m]	${\hat{\xi }}_{i}$ [m]	$\sigma_{{\hat{\xi }}_{i}}$ [mm]
α	37.6	37.6301	0.2
β	23.5	23.4885	0.2
γ	194.0	194.5904	1.6
$r_{1}$	5.0	5.0131	0.6
$r_{2}$	5.0	5.0166	0.6
$r_{3}$	5.0	5.0113	0.6
$r_{4}$	5.0	5.0112	0.6
$r_{5}$	3.8	3.8792	0.6
$r_{6}$	3.8	3.8801	0.6
$r_{7}$	3.8	3.8853	0.7
$r_{8}$	4.7	4.7489	0.7
$r_{9}$	4.7	4.7519	0.6
$r_{10}$	5.1	5.1275	0.6
$r_{11}$	5.1	5.1323	0.6
$c_{1}$	4.0	4.2939	0.5
$c_{2}$	4.0	4.2973	0.6

## Data Availability

Not applicable.

## Abbreviations

The following abbreviations are used in this manuscript:

GHM	Gauss-Helmert Model
GNSS	Global Navigation Satellite System
IVP	InVariant Point
SLR	Satellite Laser Ranging
TLS	Total Least-Squares
VLBI	Very Long Baseline Interferometry

## Appendix A. The Complete Sphero-Conical Model

The mathematical model proposed in this paper is composed of two equations: (a) one that pertains to a full circle in a horizontal plane due to azimuthal rotation, which corresponds to the cross-section of the sphere, and (b) another that explains the slant height of a cone due to rotations within the vertical planes. As described earlier, all the target points formed via horizontal and/or vertical rotations are directly connected to the IVP. Thus, the two equations of primary interest can fully describe the motion of the VLBI antenna, that is, the azimuthal rotation of target *k* (Equation (A1)) and the motion in elevation for target *l* (Equation (A2)), which are summarized as follows:

(A1) $$R_{k}\left(\alpha ,\beta ,r_{k}\right)={\left(x_{i}-\alpha \right)}^{2}+{\left(y_{i}-\beta \right)}^{2}-r_{k}^{2}=0,$$

(A2) $$C_{l}\left(\alpha ,\beta ,\gamma ,c_{l}\right)={\left(x_{i}-\alpha \right)}^{2}+{\left(y_{i}-\beta \right)}^{2}+{\left(z_{i}-\gamma \right)}^{2}-c_{l}^{2}=0,$$

where (α,β,γ) are the coordinates of the IVP that are the primary unknowns, $r_{k}$ is the radius of the horizontal circle traced by target *k*, and $c_{l}$ is the slant height of a cone traced by the vertical movement of target *l* with the apex at the IVP. The coordinates $\left(x_{i},y_{i},z_{i}\right)$ in the 3D space are simultaneously estimated using TLS, as described below.

## Appendix B. Estimation of the IVP within the Gauss-Helmert Model

Assuming the “true” coordinates for point $\left(x_{i},y_{i},z_{i}\right)$ be ($\mu_{x},\mu_{y},\mu_{z}$), then the *i*-th pair of observed variables ($x_{i},y_{i},z_{i}$) (i=1⋯n) is given by [16,18,31]

(A3) $$\begin{array}{cccc} x_{i} & =\mu_{x_{i}}+e_{x_{i}}, & E\{e_{x_{i}}\} & =0\\ y_{i} & =\mu_{y_{i}}+e_{y_{i}}, & E\{e_{y_{i}}\} & =0\\ z_{i} & =\mu_{z_{i}}+e_{z_{i}}, & E\{e_{z_{i}}\} & =0. \end{array}$$

If the random errors in the data are independent and identically distributed, the stochastic model of the random error vector can be represented as:

(A4) $$\underset{3n\times 1}{e}:=\left[\begin{array}{c} e_{x}\\ e_{y}\\ e_{z} \end{array}\right]∼\left(\left[\begin{array}{c} 0\\ 0\\ 0 \end{array}\right],\sigma_{0}^{2}\left[\begin{array}{ccc} I_{n} & 0 & 0\\ 0 & I_{n} & 0\\ 0 & 0 & I_{n} \end{array}\right]=\sigma_{0}^{2}P^{-1}\right),$$

where $\sigma_{0}^{2}$ is the unitless variance component, *P* is a 3n×3n weight matrix, and $I_{n}$ is an n×n identity matrix. In the case of the vertical motion given in Equation (A2), a (nonlinear) function that relates the *i*-th pair of variables to the unknown, nonrandom parameters ($\alpha ,\beta ,\gamma ,c_{l}$) is provided by

(A5) $$b_{i}\left(\alpha ,\beta ,\gamma ,c_{l},\mu_{x_{i}},\mu_{y_{i}},\mu_{z_{i}}\right)={\left(\mu_{x_{i}}-\alpha \right)}^{2}+{\left(\mu_{y_{i}}-\beta \right)}^{2}+{\left(\mu_{z_{i}}-\gamma \right)}^{2}-c_{l}^{2}=0.$$

The function can be linearized about the approximate expansion point ($\alpha^{0},\beta^{0},\gamma^{0},\mu_{x_{i}}^{0}$, $\mu_{y_{i}}^{0},\mu_{z_{i}}^{0}$), resulting in partial derivatives for point $\left(x_{i},y_{i},z_{i}\right)$ of the target *k* (azimuthal rotation) or target *l* (vertical rotation):

(A6) $$\begin{array}{cc} b_{i}^{0} & +2\left(\mu_{x_{i}}^{0}-\alpha^{0}\right)d\mu_{x_{i}}+2\left(\mu_{y_{i}}^{0}-\beta^{0}\right)d\mu_{y_{i}}+2\left(\mu_{z_{i}}^{0}-\gamma^{0}\right)d\mu_{z_{i}}\\ \; & -2\left(\mu_{x_{i}}^{0}-\alpha^{0}\right)d\alpha -2\left(\mu_{y_{i}}^{0}-\beta^{0}\right)d\beta -2\left(\mu_{z_{i}}^{0}-\gamma^{0}\right)d\gamma -2c_{l}^{0}dc_{l}=0, \end{array}$$

where $b_{i}^{0}$ represents the function evaluation using the initial approximation. From Equation (A3), let the differentials $d\mu_{x_{i}}$, $d\mu_{y_{i}}$, and $d\mu_{z_{i}}$ be written as

(A7) $$\begin{array}{cc} d\mu_{x_{i}} & =\mu_{x_{i}}-\mu_{x_{i}}^{0}=\left(x_{i}-\mu_{x_{i}}^{0}\right)-e_{x_{i}}\\ d\mu_{y_{i}} & =\mu_{y_{i}}-\mu_{y_{i}}^{0}=\left(y_{i}-\mu_{y_{i}}^{0}\right)-e_{y_{i}}\\ d\mu_{z_{i}} & =\mu_{z_{i}}-\mu_{z_{i}}^{0}=\left(z_{i}-\mu_{z_{i}}^{0}\right)-e_{z_{i}}, \end{array}$$

thus, upon substitution, the linearized equation can be rewritten as follows:

(A8) $$\begin{array}{cc} b_{i}^{0} & +2\left(\mu_{x_{i}}^{0}-\alpha^{0}\right)\left(x_{i}-\mu_{x_{i}}^{0}\right)+2\left(\mu_{y_{i}}^{0}-\beta^{0}\right)\left(y_{i}-\mu_{y_{i}}^{0}\right)+2\left(\mu_{z_{i}}^{0}-\gamma^{0}\right)\left(z_{i}-\mu_{z_{i}}^{0}\right)\\ \; & -2\left(\mu_{x_{i}}^{0}-\alpha^{0}\right)e_{x_{i}}-2\left(\mu_{y_{i}}^{0}-\beta^{0}\right)e_{y_{i}}-2\left(\mu_{z_{i}}^{0}-\gamma^{0}\right)e_{z_{i}}\\ \; & -2\left(\mu_{x_{i}}^{0}-\alpha^{0}\right)d\alpha -2\left(\mu_{y_{i}}^{0}-\beta^{0}\right)d\beta -2\left(\mu_{z_{i}}^{0}-\gamma^{0}\right)d\gamma -2c_{l}^{0}dc_{l}=0. \end{array}$$

Finally, we arrive at the equivalent form by rearranging

(A9) $$\begin{array}{cc} \; & \underset{w_{i}}{\underset{︸}{b_{i}^{0}+2\left(\mu_{x_{i}}^{0}-\alpha^{0}\right)\left(x_{i}-\mu_{x_{i}}^{0}\right)+2\left(\mu_{y_{i}}^{0}-\beta^{0}\right)\left(y_{i}-\mu_{y_{i}}^{0}\right)+2\left(\mu_{z_{i}}^{0}-\gamma^{0}\right)\left(z_{i}-\mu_{z_{i}}^{0}\right)}}\\ \; & \;-\underset{B_{i}}{\underset{︸}{\left[\begin{array}{ccc} 2(\mu_{x_{i}}^{0}-\alpha^{0}) & 2(\mu_{y_{i}}^{0}-\beta^{0}) & 2(\mu_{z_{i}}^{0}-\gamma^{0}) \end{array}\right]}}\underset{e_{i}}{\underset{︸}{\left[\begin{array}{c} e_{x_{i}}\\ e_{y_{i}}\\ e_{z_{i}} \end{array}\right]}}\\ \; & \;-\underset{A_{i}}{\underset{︸}{\left[\begin{array}{cccc} 2(\mu_{x_{i}}^{0}-\alpha^{0}) & 2(\mu_{y_{i}}^{0}-\beta^{0}) & 2(\mu_{z_{i}}^{0}-\gamma^{0}) & 2r^{0} \end{array}\right]}}\underset{\xi }{\underset{︸}{\left[\begin{array}{c} d\alpha \\ d\beta \\ d\gamma \\ dc_{l} \end{array}\right]}}=0, \end{array}$$

or, more compactly,

(A10) $$w_{i}=A_{i}\xi +B_{i}e_{i}\;\left(i=1\cdots n_{l}\right).$$

Similarly, the mathematical model for the horizontal rotation can be represented by

(A11) $$\begin{array}{cc} a_{i}^{0} & +2\left(\mu_{x_{i}}^{0}-\alpha^{0}\right)\left(x_{i}-\mu_{x_{i}}^{0}\right)+2\left(\mu_{y_{i}}^{0}-\beta^{0}\right)\left(y_{i}-\mu_{y_{i}}^{0}\right)\\ \; & -\left[\begin{array}{ccc} 2(\mu_{x_{i}}^{0}-\alpha^{0}) & 2(\mu_{y_{i}}^{0}-\beta^{0}) & 0 \end{array}\right]\left[\begin{array}{c} e_{x_{i}}\\ e_{y_{i}}\\ e_{z_{i}} \end{array}\right]\\ \; & -\left[\begin{array}{cccc} 2(\mu_{x_{i}}^{0}-\alpha^{0}) & 2(\mu_{y_{i}}^{0}-\beta^{0}) & 0 & 2r^{0} \end{array}\right]\left[\begin{array}{c} d\alpha \\ d\beta \\ d\gamma \\ dc_{l} \end{array}\right]=0. \end{array}$$

Assuming that a target $\left(x_{i},y_{i},z_{i}\right)$ is involved in both the azimuthal rotation ($r_{k}$) and the vertical motion ($c_{l}$), we arrive at the estimation model for the TLS within the GHM by combining Equations (A9) and (A11):

(A12) $$w_{i}=A_{i}\xi +B_{i}e_{i}\;.$$

The (residual) observation vector is given by

(A13) $$w_{i}={\left[\begin{array}{cc} a_{i} & b_{i} \end{array}\right]}^{T},$$

where

(A14) $$\begin{array}{cc} a_{i} & ={\left(x_{i}^{0}-\alpha^{0}\right)}^{2}+{\left(y_{i}^{0}-\beta^{0}\right)}^{2}-{\left(r_{k}^{0}\right)}^{2}\\ \; & +2\left(\mu_{x_{i}}^{0}-\alpha^{0}\right)\left(x_{i}-\mu_{x_{i}}^{0}\right)+2\left(\mu_{y_{i}}^{0}-\beta^{0}\right)\left(y_{i}-\mu_{y_{i}}^{0}\right),\\ b_{i} & ={\left(x_{i}^{0}-\alpha^{0}\right)}^{2}+{\left(y_{i}^{0}-\beta^{0}\right)}^{2}+{\left(z_{i}^{0}-\gamma^{0}\right)}^{2}-{\left(c_{l}^{0}\right)}^{2}\\ \; & +2\left(\mu_{x_{i}}^{0}-\alpha^{0}\right)\left(x_{i}-\mu_{x_{i}}^{0}\right)+2\left(\mu_{y_{i}}^{0}-\beta^{0}\right)\left(y_{i}-\mu_{y_{i}}^{0}\right)+2\left(\mu_{z_{i}}^{0}-\gamma^{0}\right)\left(z_{i}-\mu_{z_{i}}^{0}\right), \end{array}$$

and the corresponding observation error and the design matrix for the unknown parameters are represented by

(A15) $$\begin{array}{cc} B_{i}\cdot e_{i} & =\left[\begin{array}{ccc} 2(\mu_{x_{i}}^{0}-\alpha^{0}) & 2(\mu_{y_{i}}^{0}-\beta^{0}) & 0\\ 2(\mu_{x_{i}}^{0}-\alpha^{0}) & 2(\mu_{y_{i}}^{0}-\beta^{0}) & 2(\mu_{z_{i}}^{0}-\gamma^{0}) \end{array}\right]\left[\begin{array}{c} e_{x_{i}}\\ e_{y_{i}}\\ e_{z_{i}} \end{array}\right] \end{array}$$

(A16) $$\begin{array}{cc} A_{i}\cdot \xi & =\left[\begin{array}{ccccc} 2(\mu_{x_{i}}^{0}-\alpha^{0}) & 2(\mu_{y_{i}}^{0}-\beta^{0}) & 0 & 2r_{k}^{0} & 0\\ 2(\mu_{x_{i}}^{0}-\alpha^{0}) & 2(\mu_{y_{i}}^{0}-\beta^{0}) & 2(\mu_{z_{i}}^{0}-\gamma^{0}) & 0 & 2c_{l}^{0} \end{array}\right]\left[\begin{array}{c} d\alpha \\ d\beta \\ d\gamma \\ dr_{k}\\ dc_{l} \end{array}\right]. \end{array}$$

The least-squares solution (LESS) of the GHM produces an estimate of the unknown parameters ($\hat{\xi }$) and a prediction of the unknown random errors, that is, residuals ($\tilde{e}$) as follows:

(A17) $$\hat{\xi }={\left[A^{T}{\left(BP^{-1}B^{T}\right)}^{-1}A\right]}^{-1}A^{T}{\left(BP^{-1}B^{T}\right)}^{-1}w$$

(A18) $$\tilde{e}=P^{-1}B^{T}{\left(BP^{-1}B^{T}\right)}^{-1}\left(w-A\;\hat{\xi }\right).$$

The corresponding estimated dispersion matrix for the estimated parameters and the estimated variance component are given by

(A19) $${\hat{\sigma }}_{0}^{2}=\frac{{\left(B\;\tilde{e}\right)}^{T}{\left(BP^{-1}B^{T}\right)}^{-1}\left(B\;\tilde{e}\right)}{r}=\frac{{\tilde{e}}^{T}P\;\tilde{e}}{r}$$

(A20) $$\hat{D}\left\{\hat{\xi }\right\}={\hat{\sigma }}_{0}^{2}{\left[A^{T}{\left(BP^{-1}B^{T}\right)}^{-1}A\right]}^{-1},$$

where r:=rkB−rkA is the redundancy of the model.
